# Increased neural reactivity to emotional pictures in men with high hair testosterone concentrations

**DOI:** 10.1093/scan/nsz067

**Published:** 2019-09-11

**Authors:** Sanja Klein, Onno Kruse, Isabell Tapia León, Tobias Stalder, Rudolf Stark, Tim Klucken

**Affiliations:** 1 Department of Psychotherapy and Systems Neuroscience, Justus Liebig University, Giessen 35394, Germany; 2 Bender Institute for Neuroimaging (BION), Justus Liebig University, Giessen 35394, Germany; 3 Clinical Psychology, University Siegen, Siegen 57076, Germany

**Keywords:** fMRI, emotion, testosterone

## Abstract

Testosterone has been linked to alterations in the activity of emotion neurocircuitry including amygdala, orbitofrontal cortex (OFC) and insula and diminished functional amygdala/prefrontal coupling. Such associations have only ever been studied using acute measures of testosterone, thus little is known about respective relationships with long-term testosterone secretion. Here, we examine associations between hair testosterone concentration (HTC), an index of long-term cumulative testosterone levels and neural reactivity during an emotional passive viewing task using functional magnetic resonance imaging (fMRI). Forty-six men viewed negative, positive and neutral pictures in the MRI. HTCs were assessed from 2 cm hair segments. The emotional paradigm elicited neural activation in the amygdala, insula and OFC. HTCs were associated with increased reactivity to negative pictures in the insula and increased reactivity to positive pictures in the OFC. We show an association of long-term testosterone levels with increased emotional reactivity in the brain. These results suggest a heightened emotional vigilance in individuals with high trait testosterone levels.

## Introduction

Testosterone and other androgens have been shown to have neuroregulatory effects on serotonergic and dopaminergic systems by binding to androgen receptors in the amygdala, striatum and hypothalamus (Rubinow and Schmidt, 1996). These systems play an important role in emotion and behavior regulation as well as in general emotion processing in many mammals including humans. Therefore, testosterone is frequently examined as a modulator of these functions and associated psychopathologies (Rosell and Siever, 2015).

Such experimental works on the links between testosterone and human emotion processing has revealed exogenous testosterone to reduce skin conductance responses to negative pictures (Hermans *et al.*, 2007) as well as fear potentiation of the startle reflex (Hermans *et al.*, 2006). In line with this, Van Honk *et al.* (2005) found a lower fear-related attentional bias in a masked emotional Stroop task after testosterone administration. While many studies have focused on acute testosterone secretion or administration, little is known about the role of long-term cumulative testosterone. Testosterone measures aggregated over many saliva samples taken on multiple different days have been shown to be a good predictor of emotion regulation problems (Granger *et al.*, 2003; van Bokhoven *et al.*, 2006). Notably, Granger *et al.* (2003) found that these aggregated testosterone measures were superior in predicting emotion regulation problems compared to single-state measures. In a recent study, Grotzinger *et al.* (2018) utilized hair analyses to investigate long-term cumulative testosterone levels alongside short-term salivary testosterone data. Importantly, they found dysfunctional emotion regulation linked with hair testosterone concentrations (HTCs) but not with acute salivary testosterone levels, which corresponds to the notion that long-term endocrine data might be better suited for capturing associations with stable, trait-like behavioral dispositions. The method of hair steroid analysis, which was used in this research, has increasingly gained acceptance over the past decade for providing a reliable, valid and robust index of long-term hormone secretion patterns (reviews: Stalder *et al.*, 2017; Stalder and Kirschbaum, 2012). While a lot of research exists using hair cortisol concentrations, so far, no comparable studies using hair testosterone analysis have been conducted to investigate links with general emotion processing.

Most studies concerned with acute testosterone have focused on fear-related stimuli, but more recently Bos *et al.* (2012) proposed a model featuring testosterone as important for helping to detect and cope with not only potentially threatening but also rewarding stimuli by simultaneously increasing salience perception and reducing fear to prepare for action. Thus, testosterone should be associated with increased activity in emotion processing regions when viewing negative as well as positive emotionally salient stimuli.

Accordingly, high acute testosterone levels have been linked with increased neural reactivity to emotional stimuli. In functional magnetic resonance imaging (fMRI) studies, endogenous and exogenous testosterone have been linked to increased activations in the amygdala (van Wingen *et al.*, 2011; Heany *et al.*, 2016) and the orbitofrontal cortex (OFC; van Wingen *et al.*, 2011) in response to potentially threatening and rewarding stimuli. Specifically, increased amygdala activity correlating with high testosterone levels was found in response to negative (Hermans *et al.*, 2008; Derntl *et al.*, 2009; Manuck *et al.*, 2010) as well as positive pictures (van Wingen *et al.*, 2009; Bos *et al.*, 2013) and emotional sounds (Bos *et al.*, 2010). High testosterone levels have also been linked with increased reactivity to negative faces in prefrontal regions, in particular the OFC (Hermans *et al.*, 2008) and the ventromedial prefrontal cortex ( Stanton *et al.*, 2009). The insula as an important part of the salience network (Menon and Uddin, 2010; Uddin, 2015) has mostly been neglected in emotion processing studies concerned with testosterone. Bos *et al.* (2010) found that testosterone increased neural response of the insula to emotional sounds.

This study provides the first investigation of the relationship between HTC and emotion processing neuroimaging data. We hypothesized that subjects with higher HTC show increased salience perception of emotional pictures independent of valence compared to neutral pictures in all response systems measured. Thus, we hypothesized that subjects with higher HTC levels show higher subjective salience ratings, increased electrodermal activity and increased hemodynamic responses in emotion processing and saliency areas (amygdala, OFC and insula) when viewing emotional pictures compared to neutral.

## Materials and methods

### Subjects

Participants were 46 males (age: M = 23.35 SD = 3.00 years), most of them students. All subjects were right handed, were German native speakers and had normal or corrected-to-normal vision. Past or current mental or neurological problems, consumption of psychotropic drugs, chronic illnesses or treatments and conditions preventing them from entering the MRI scanner were exclusion criteria. The study was conducted in accordance with the Declaration of Helsinki (2008) and approved by the local ethics committee.

### Procedure

We used a picture perception design with blocks of emotional pictures as stimuli. For a detailed description of the picture perception paradigm see Wehrum *et al.* (2013). For this, 30 pictures each in 4 emotional categories (positive, negative, neutral and sexual) were taken from the International Affective Picture System (Lang *et al.*, 2008) and the internet. One block consisted of the sequential presentation of five pictures of the same emotional category. Each picture was shown for 3 s. Participants passively viewed the alternating blocks of the four emotional categories. After each block, subjects rated the pictures on three scales (see Subjective ratings). The experiment ran for a total of 15.5 min, starting and ending with the presentation of a white fixation cross for 37.5 s. The paradigm includes sexual stimuli because it is used in a variety of studies from this group, but for our particular hypotheses, they were not relevant and therefore were excluded from analysis. Stimuli were presented using Presentation® software (Version 17.0, Neurobehavioral Systems, Inc., Berkeley, CA, www.neurobs.com) on a monitor at the back of the scanner (resolution: 1920 × 1080 pixels; BOLDscreen 32, Cambridge Research Systems) that the subjects viewed through a mirror mounted on the head coil (visual angle 28 degrees).

### Hair testosterone analysis

Hair strands with a diameter of ∼3 mm were cut from the posterior vertex region as close to the scalp as possible using hair scissors. Hair samples were stored in aluminum foil for protection until analysis. Testosterone concentrations were determined from the 2 cm hair segment closest to the scalp. Based on a hair growth rate of ∼1 cm/month (Wennig, 2000), these segments are assumed to reflect hair grown over the 2 month period prior to the respective sampling points. Testosterone concentration was measured using liquid chromatography tandem mass spectrometry (LC–MS/MS; (Gao *et al.*, 2016) according to a published protocol (Gao *et al.*, , 2013). HTC data in picogram per milligram were then correlated (non-parametric) with the blood–oxygen level-dependent (BOLD) response in the group-level fMRI analysis as well as with picture ratings and skin conductance data using SPSS 22 (IBM Corp., 2013).

### Subjective ratings

After each presented picture block, subjects rated valence, arousal and sexual arousal on a 9-point Likert-type scale. All scales ranged from 0 (indicating ‘not arousing at all’, ‘very unpleasant’ or ‘not sexually arousing at all’) to 8 (indicating ‘very arousing’, ‘very pleasant’ or ‘very sexually arousing’). As the sexual arousal ratings corresponded to the sexual pictures, they were not relevant to our hypothesis and therefore were not included in the analysis. Rating scores were later averaged over subjects and analyzed using SPSS 22 (IBM Corp., 2013). For each picture category, mean arousal and valence ratings were computed. Mean ratings of negative and positive pictures were analyzed in two analyses of variance (ANOVAs) with one emotion factor (negative, positive and neutral). Significant effects were followed up with paired *t* tests and were Bonferroni-corrected for multiple comparisons (corrected significance threshold was α = 0.02 for all follow-up tests). Rating analyses for the sexual pictures are reported in the supplement.

### Skin conductance

Skin conductance was measured during the picture presentation with reusable Ag/AgCl electrodes filled with isotonic (0.05 M NaCl) electrolyte medium placed on the non-dominant left hand. Data were collected with a sampling rate of 1 kHz. For preprocessing and data analysis, Ledalab 3.4.4 was used (Benedek and Kaernbach, 2010). First, the data were downsampled to 100 Hz and smoothed with a 32 sample full width at half maximum (FWHM) Gaussian kernel. One subject had to be excluded due to missing data caused by technical difficulties, leaving a sample of 45 subjects for skin conductance response (SCR) analysis. As each picture was presented for 3 s, this time window was defined as analysis window. The extracted response was defined as the largest difference between a maximum and the minimum that directly preceded it. The preceding minimum had to be within the analysis window for the response to be counted. Responses smaller than 0.01 μS were considered zero responses. All maximum responses were log (μS + 1) transformed to correct for violation of normal distribution of the data. Mean SCRs for each picture block were calculated subsequently. Skin conductance data for the different emotional categories were then averaged over all blocks and analyzed in an ANOVA with one emotion factor (negative, positive or neutral). Significant effects were followed up with paired *t* tests and were Bonferroni-corrected for multiple comparisons (corrected significance threshold was α = 0.02 for all follow-up tests). Skin conductance analyses for the sexual pictures are reported in the supplement.

### fMRI data acquisition and analysis

All MRI images were acquired using a 3 Tesla whole-body tomograph (Siemens Prisma) with a 64-channel head coil. The structural images consisted of 176 T1-weighted sagittal slices (slice thickness, 0.9 mm; FoV = 240 mm; TR = 1.58 s; TE = 2.3 s). For the functional images, a total of 420 images was acquired with a T2*-weighted gradient echo-planar imaging (EPI) with 36 slices covering the whole brain (voxel size = 3 × 3 × 3.5 mm; gap = 0.5 mm; descending slice acquisition; TR = 2 s; TE = 30 ms; flip angle = 75 degrees; FoV = 192 × 192 mm^2^; matrix size = 64 × 64; GRAPPA = 2). The field of view was positioned automatically relative to the AC-PC line with an orientation of −40 degrees. Preprocessing, first- and second-level analyses were done using SPM 12 (Wellcome Department of Cognitive Neurology, 2014) implemented in MATLAB (The MathWorks Inc., 2012).

For preprocessing, all anatomical images were coregistered to an MNI (Montreal Neurological Institute) template and segmented. All EPI images were coregistered to the anatomical images, realigned and unwarped, slice time corrected, normalized to MNI standard space and smoothed with a Gaussian kernel at 6 mm FWHM. Functional data were analyzed for outlying volumes using a distribution free approach for skewed data (Schweckendiek *et al.*, 2013). Each resulting outlying volume was later modeled within the general linear model as a regressor of no interest. The picture categories were negative, positive, sexual and neutral. Each category was modeled as a regressor of interest. The rating phases following the picture blocks were modeled as a regressor of no interest. All regressors were convolved with the canonical hemodynamic response function. Six movement parameters were entered as covariates alongside regressors of no interest for the identified outlying volumes. The time series was then filtered with a high pass filter (time constant = 128 s). On the group level, three contrasts comparing emotional with neutral pictures were examined: negative–neutral, positive–neutral and emotional–neutral. The emotional regressor consisted of both negative and positive regressors. One-sample *t* tests as well as multiple regressions with HTC as a predictor were performed for the three contrasts. As an exploratory analysis, we also examined the contrast negative–positive. Additionally, the same analyses were computed for the sexual–neutral contrast and reported in the supplement.

Because the HTC data contained outliers (see Supplementary Figure 1 for histogram), statistical inference in second-level analyses was based on non-parametric permutation tests using threshold-free cluster enhancement (TFCE) with 5000 permutations (Nichols and Holmes, 2002; Smith and Nichols, 2009). Whole brain analyses on the voxel level were conducted with *P* < 0.05 family-wise error (FWE) corrected. Region of interest (ROI) analyses on the voxel level were conducted using small volume correction with *P* < 0.05 (FWE). Amygdala, OFC and insula were chosen as ROIs because they have been previously reported in studies concerned with emotion processing and testosterone (Bos *et al.*, 2010; van Wingen *et al.*, 2011; Heany *et al.*, 2016). The ROI masks for amygdala and insula were taken from the Harvard Oxford Cortical Atlas (HOC; both threshold 50). The mask for OFC was created in MARINA (Walter *et al.*, 2003) because no OFC mask is available in the HOC.

**Fig. 1:**
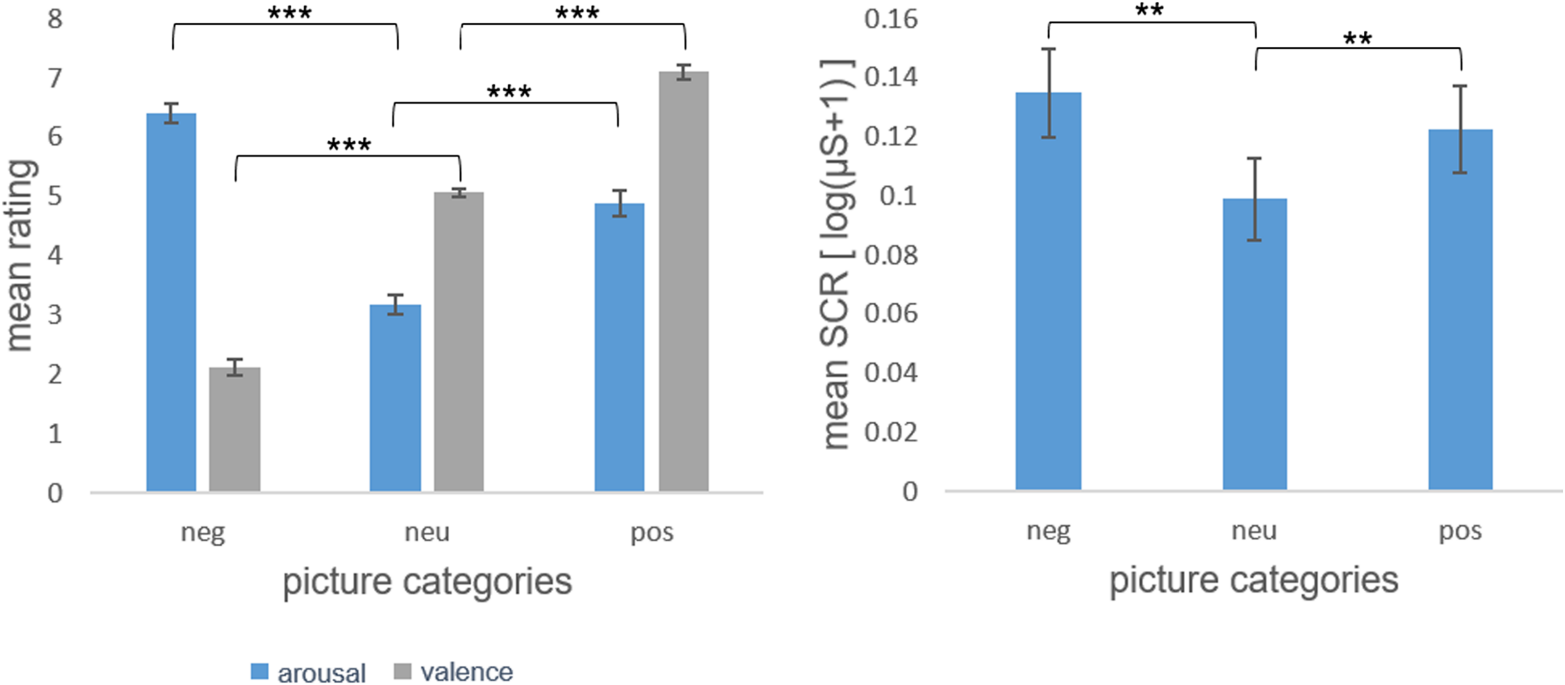
Mean valence and arousal ratings of emotional pictures by category (neg: negative, neu: neutral, pos: positive) on the left. Standard errors of means are given as error indicators. ^***^significant at p<.001. Mean skin conductance response to negative, neutral and positive pictures on the right. Standard errors of means are given as error indicators. ^**^significant at p<.01.

## Results

Mean HTC was *M* = 1.39 (SD = 1.16), MED = 1.05. For the mean arousal ratings, the ANOVA revealed a significant main effect of emotion [*F*(2) = 120.33, *P* < 0.001]. Mean arousal ratings were higher for both negative [*t*(45) = 13.57, *P* < 0.001] and positive [*t*(45) = 8.12, *P* < 0.001] pictures compared to neutral pictures. Mean arousal ratings were also higher for negative pictures compared to positive [*t*(45) = 6.84, *P* < 0.001]. For the mean valence ratings, the ANOVA revealed a significant main effect of emotion as well [*F*(1.50) = 419.83, *P* < 0.001, Greenhouse–Geisser-corrected]. As expected, valence ratings were lower for negative [*t*(45) = −19.69, *P* < 0.001] and higher for positive [*t*(45) = 16.05, *P* < 0.001] pictures compared to neutral. Unsurprisingly, mean valence ratings were higher for positive pictures compared to negative [*t*(45) = 23.732, *P* < 0.001]. No significant correlation between HTC and subjective rating data was found (all *P* > 0.05). For the skin conductance data, we also found a main effect of emotion [*F*(2) = 6.48, *P* = 0.002]. Mean skin conductance was higher during negative [*t*(44) = 3.62, *P* = 0.001] and positive [*t*(44) = 2.88, *P* = 0.006] picture blocks compared to neutral picture blocks. Mean skin conductance did not differ between negative and positive picture blocks [*t*(44) = 1.09, *P* = 0.280]. No correlation of skin conductance and HTC was found (all *P* > 0.05) Subjective rating and SCR results are displayed in Figure 1.

### Hemodynamic responses

#### Emotion processing

Positive pictures as compared to neutral elicited a BOLD response in bilateral OFC and insula. Negative pictures compared to neutral were associated with increased BOLD responses in the bilateral amygdala in addition to OFC and insula (see Table 1).

**Table 1:** Peak voxels in respective ROIs of the BOLD contrasts (one-sample *t* tests) with cluster size (*k*) and statistics (FWE-corrected)

Contrast	Structure	Side	*x*	*y*	*z*	*k*	*t*_max_	*P*_corr_
Negative–neutral	Amygdala	L	−22	−6	−12	239	8.93	<0.001
		R	20	−4	−12	260	8.07	<0.001
	OFC	L	−2	54	−16	735	4.86	0.003
		R/L	0	54	−18	415	4.61	0.006
	Insula	L	−32	10	−12	706	8.31	<0.001
		R	38	6	−10	607	6.25	<0.001
Positive–neutral	OFC	R	14	30	−16	56	3.98	0.036
	Insula	L	−42	14	−4	493	4.74	0.004
Negative–positive	Amygdala	L	−22	−8	−12	226	8.37	<0.001
		R	20	−4	−12	200	10.42	<0.001
	OFC	L	−2	50	−16	686	5.24	0.001
		R/L	0	50	−16	694	5.35	<0.001
	Insula	L	−40	−6	−2	545	6.39	<0.001
		R	40	−4	−2	482	5.52	<0.001
Positive–negative	Insula	L	−36	−18	18	8	3.80	0.042

**Table 2:** Peak voxels in respective ROIs of the testosterone regression analyses with equivalent cluster size (equivk) and TFCE statistics (FWE-corrected)

Contrast	Structure	Side	*x*	*y*	*z*	equivk	**TFCE**	*P*_Corr_
Negative–neutral	Insula	R	36	10	−10	14	112.59	0.020
Positive–neutral	OFC	L	−2	56	−2	33	723.59	0.041
		R	4	48	−6	75	730.30	0.043

#### Emotion processing and hair testosterone

**Fig. 2:**
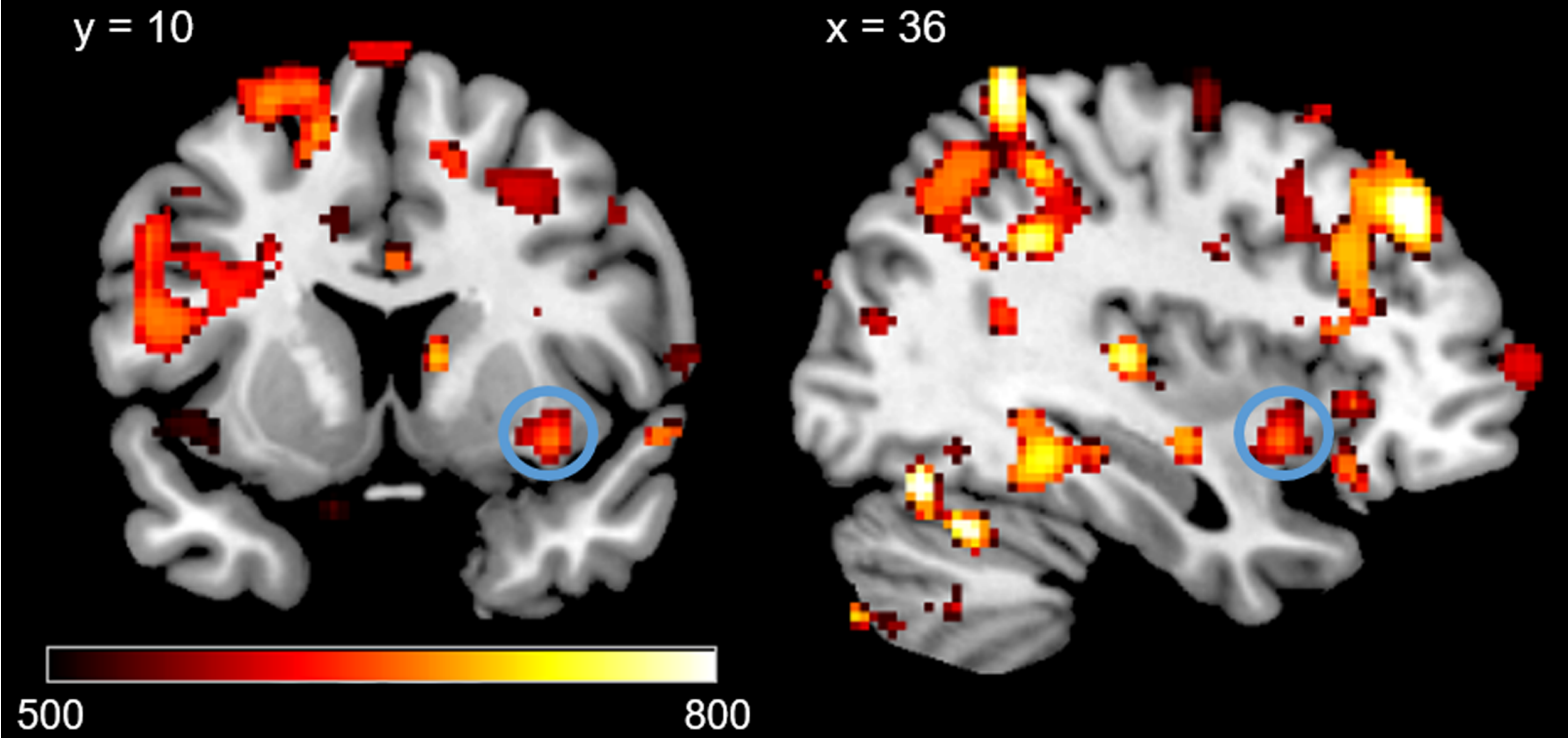
Positive correlations between HTC and significant ROI activations during negative pictures – neutral pictures on voxel level P < 0.05 (FWE-corrected) in the right insula. Displayed TFCE-values are thresholded at TFCE > 500.

**Fig. 3:**
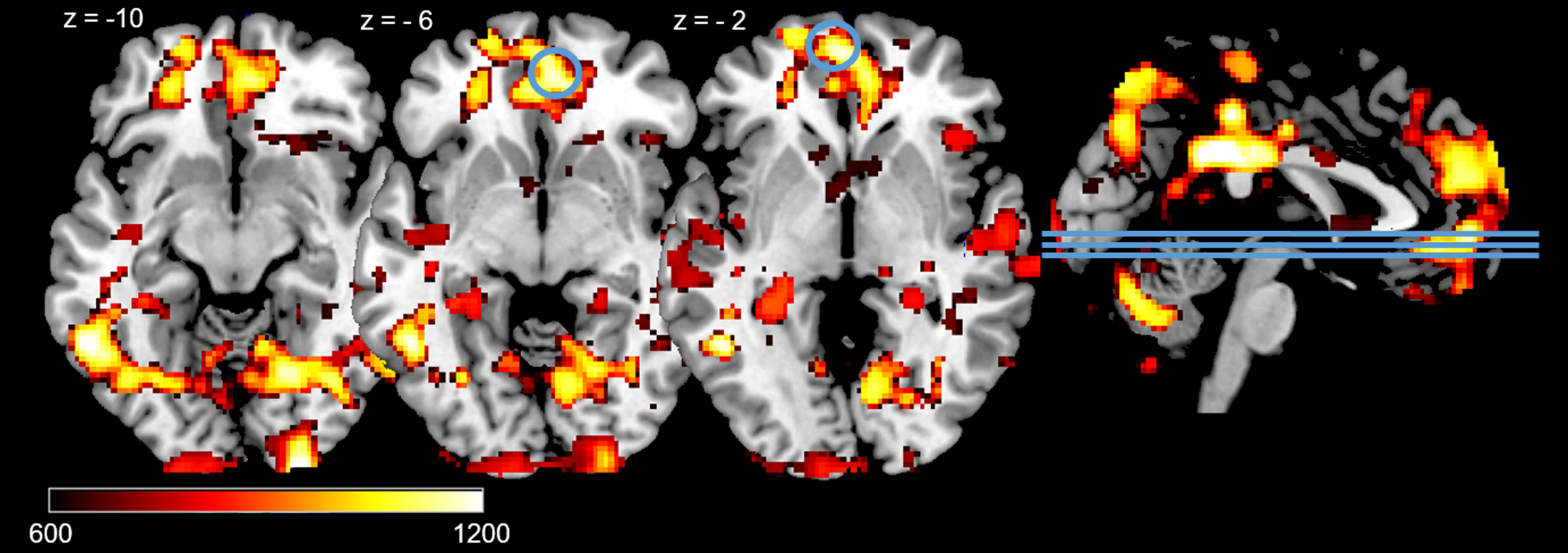
Positive correlations between HTC and significant ROI activations during positive pictures (positive - neutral) on voxel level P< 0.05 (FWE-corrected) in right and left OFC. Lines on the sagittal slices on the right side indicate the axial slices depicted on the left. Displayed TFCE-values are thresholded at TFCE > 600.

The results showed that high HTC correlated with BOLD responses in the right insula during the processing of negative pictures (negative–neutral; see Table 2, Figure 2). In addition, high HTC was linked with increased BOLD responses in the left and right OFC during the processing of positive pictures (positive–neutral; see Table 2, Figure 3). No whole-brain or ROI results were found in the negative–positive/positive–negative contrast. Whole-brain results for the negative–neutral and positive–neutral contrasts are reported in the supplement (Table 1).

## Discussion

This study provides the first look at the relation between long-term cumulative testosterone levels (HTC) and neural emotion processing. The picture perception paradigm showed blocks of negative, positive and neutral pictures. We found increased brain reactivity to negative and positive emotional pictures in the OFC and insula but not in the amygdala in subjects with high HTC.

The correlation of HTC and neural activation in the OFC in response to positive pictures supports current models of testosterone (Bos *et al.*, 2012) and extends other findings observing the effects of acute (Stanton *et al.*, 2009) testosterone in frontal regions to long-term cumulative hormone levels. A connection of exogenous testosterone and emotional reactivity in the insula has been found in past research for women listening to emotional sounds (Bos *et al.*, 2010). The association we found between HTC and insula reactivity to negative pictures extends the evidence to men with high long-term testosterone levels and visual emotional stimuli. The OFC as well as the insula have been implicated in the processing of social emotional information (Singer *et al.*, 2004; Goodkind *et al.*, 2012) and most testosterone effects are found in such social-emotional contexts (Heany *et al.*, 2016). Long-term high testosterone output could lead to these regions being more sensitive to such emotionally salient environment stimuli, making individuals experience more anger if the environment is threatening or more positive affect if the environment is rewarding. This altered emotional processing, in turn, could influence behavior and explain why testosterone is often associated with not only aggressive behavior and emotion regulation problems (van Bokhoven *et al.*, 2006; Grotzinger *et al.*, 2018) but also increased sexual behavior, positive mood and reward sensitivity (van Anders *et al.*, 2007; Hermans *et al.*, 2010; Amanatkar *et al.*, 2014).

We also found significant (FWE-corrected *P*_TFCE_ < 0.05) associations of HTC with neural activations in exploratory whole brain analyses. Mainly, HTC was linked to activations in frontal, limbic and paralimbic structures as well as visual processing regions during negative and positive picture presentation compared to neutral with no significant differences between valence categories. Specifically, we found increased reactivity to negative pictures in the superior parietal lobule and the anterior supramarginal gyrus. These regions are anatomically part of the premotor cortex (Nachev *et al.*, 2008), which is associated with motor control and response inhibition in conflict situations (Nachev *et al.*, 2007). We also found increased reactivity in the anterior cingulate in response to positive pictures. The anterior cingulate cortex is associated with response inhibition, cognitive control and conflict monitoring as well (Botvinick *et al.*, 2001). Thus, activation in these regions might reflect a greater need to inhibit behavioral responses or response motivation to emotional stimuli in high HTC individuals. Additionally, increased activation of areas associated with visual perception and attention like the lingual gyrus (Fink *et al.*, 1996) and the occipital pole (Puce *et al.*, 1996; Mevorach *et al.*, 2010) are strong indicators of increased visual attention to negative and positive emotional stimuli in high HTC subjects. Taken together, the whole brain results can be regarded as another indicator for the testosterone model put forth by Bos *et al.* (2012), where testosterone is associated with increased attention and responsiveness to motivationally salient environment stimuli.

While the exact mechanisms of testosterone acting in the brain are still not completely understood, one potential explanation for the effects of the hormone on neural emotional processing is through binding to androgen receptors such as the γ-aminobutyric acid (GABA) receptor (Rubinow and Schmidt, 1996; McHenry *et al.*, 2014). In mice, the repeated administration of testosterone has anxiolytic effects, while the administration of a GABA antagonist blocks these effects (Fernández-Guasti and Martínez-Mota, 2005). In accordance with this animal model, positron emission tomography studies in humans have shown that post-traumatic stress disorder patients display decreased binding with GABA receptors in the right OFC and right insula (Malizia *et al.*, 1998) and in the prefrontal cortex (Bremner *et al.*, 2000). A deficit in GABA receptors in the OFC is also found in patients with major depression (Rajkowska *et al.*, 2007). The influence of testosterone on GABA receptors might mediate some of the hormones anxiolytic (van Honk *et al.*, 2005; Hermans *et al.*, 2006) and antidepressive (Wang, 2000; Rosen *et al.*, 2017) properties, which have been shown in humans. This model also illustrates a possible pathway of how high HTC could be associated with increased OFC and insula activity in this study through repeated binding of the hormone to the androgen receptors in those regions over time. Extending past research, which examined mostly state testosterone levels, our results can be regarded as evidence for a heightened trait vigilance and possibly better coping with environmental challenges in people with high trait testosterone output. Linking the knowledge about the hormone and its corresponding receptors, stable long-term high testosterone levels could also serve as a protective factor for mood- and anxiety-related psychopathologies.

We also examined a comparison of negative *vs* positive pictures as an exploratory analysis. While negative pictures were rated higher in arousal and recruited most emotion processing ROIs more compared to positive, this emotional valence difference did not carry through to the HTC correlations. While activation patterns differ between valence categories in association with HTC, we did not find any evidence of a significant difference between emotions in association with HTC.

The difference in arousal ratings and task effect between negative and positive pictures might be due to a possible anchoring effect caused by the sexual pictures. The sexual pictures were rated higher in arousal compared to positive pictures and recruited all emotion processing ROIs (see supplementary material). The presence of the sexual pictures might have made the positive pictures less interesting and exciting for the subjects, skewing their perception. We only see this in the ratings and the task effects, so the HTC associations with neural activation do not seem affected by this.

Interestingly, we found no correlation of HTC with amygdala reactivity to neither negative nor positive pictures. The amygdala has been reported to have a positive association with acute endogenous and exogenous testosterone during emotional tasks in some studies (van Wingen *et al.*, 2009; Manuck *et al.*, 2010; Bos *et al.*, 2013; Derntl *et al.*, 2009; Hermans *et al.*, 2008). As these studies all utilized acute hormone measures, this discrepancy could point to the amygdala being more dependent on state fluctuations of the hormone.

Since gender differences are prevalent in long-term testosterone levels (Grotzinger *et al.*, 2018), future studies should include women to compare testosterone concentrations and their effects on emotion processing between genders. Future research should also include an acute measure of testosterone to validate and compare with the hair measurement.

### Conclusion

Taken together, our results demonstrate how men with high long-term testosterone levels display increased reactivity of the emotion processing neurocircuitry to salient emotional stimuli. The positive association of the insula with testosterone when viewing negative pictures can be interpreted as increased processing of aversive emotionally salient stimuli in men with higher baseline testosterone secretion. Increased hemodynamic responses to positive pictures in the bilateral OFC suggest similar altered processing as for the negative pictures. In conclusion, we showed for the first time that long-term trait testosterone levels are associated with neural emotion processing differences.

## Funding

This study was in part supported by the German Research Foundation (298597483).

## Conflict of interest

None declared.

## Supplementary Material

scan-18-418-File005_nsz067
