# Supplementary material for: Increased neural reactivity to emotional pictures in men with high hair testosterone concentrations
Source: Soc Cogn Affect Neurosci. 2019 Sep 11;14(9):1009–16. doi: 10.1093/scan/nsz067 (PMC6917022; doi:10.1093/scan/nsz067)
Supplement: scan-18-418-File005_nsz067 [file scan-18-418-file005_nsz067.docx]

**Supplement**

**
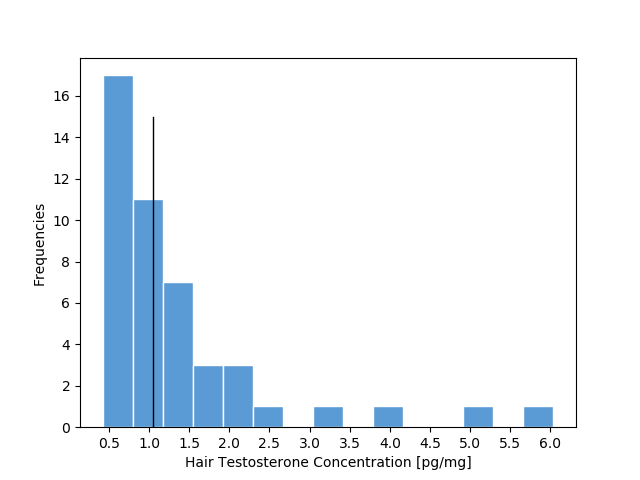
1. HTC distribution**

*Figure 1: Histogram of HTC distribution. M = 1.39 (SD = 1.16), MED = 1.05 (indicated by the black vertical line). Two values where larger than 3 × the interquartile range + the median and are thus classified as outliers.*

**2. Wholebrain HTC effects**

| *Table 1: Peak voxels of the testosterone regression analyses with equivalent cluster size (equivk) and TFCE-statistics (FWE-corrected).* | | | | | | | | |
| --- | --- | --- | --- | --- | --- | --- | --- | --- |
| Contrast | Structure | Side | x | y | z | equivk | **TFCE** | **P**_Corr_ |
| negative - neutral | anterior supramarginal gyrus | L | -28 | -40 | 34 | 368 | 1396.07 | .032 |
|  | Superior parietal lobule | L | -32 | -48 | 70 | 58 | 1299.25 | .039 |
|  | Lingual gyrus | R | 8 | -72 | -2 | 26 | 1239.80 | .045 |
| positive - neutral | posterior cingulate gyrus | R | 10 | -38 | 40 | 914 | 1871.62 | .010 |
|  | anterior cingulate gyrus | L | -4 | 14 | 36 | 4 | 1146.40 | .049 |
|  | superior frontal gyrus | R | 22 | 34 | 32 | 407 | 1531.40 | .018 |
|  |  | R | 18 | 26 | 54 | 171 | 1313.14 | .030 |
|  | inferior temporal gyrus | L | -56 | -56 | -14 | 127 | 1269.28 | .032 |
|  | lateral occipital cortex | R | 36 | -72 | 44 | 62 | 1322.27 | .029 |
|  | postcentral gyrus | L | -48 | -38 | 54 | 86 | 1252.38 | .035 |
|  | occipital pole | R | 24 | -100 | -10 | 18 | 1242.16 | .036 |
|  | precuneous cortex | L | -12 | -66 | 28 | 68 | 1218.85 | .039 |
|  |  | L | -6 | -76 | 52 | 42 | 1179.22 | .043 |
|  | occipital fusiform gyrus | R | 24 | -68 | -18 | 63 | 1194.56 | .042 |
|  | temporal occipital fusiform cortex | L | -32 | -64 | -22 | 61 | 1185.93 | .043 |
|  | paracingulate gyrus | R | 4 | 48 | -6 | 46 | 1175.73 | .045 |
|  | middle frontal gyrus | R | 28 | 16 | 44 | 14 | 1152.66 | .048 |
|  |  | R | 32 | 16 | 56 | 14 | 1146.56 | .049 |

**3. Sexual pictures**

**3.1 Ratings & Skin conductance**

Sexual pictures were rated higher in valence [*t*(45) = 8.02, *p*<.001] and arousal [*t*(45) = 10.47, *p*<.001] compared to neutral. Sexual pictures were rated higher in arousal [*t*(45) = 4.85, *p*<.001] and lower in valence [*t*(45) = -3.74, *p*=.001] compared to positive. Mean skin conductance was higher for sexual pictures compared to neutral [*t*(44) = 4.13, *p<*.001] and slightly higher compared to positive [*t*(44) = 2.03, *p* = .049] (although this would not survive a multiple comparison correction which we did not perform here **since the analysis was exploratory**). No correlations of HTC with ratings of sexual pictures or SCR during sexual pictures were found.

**3.2 fMRI Task effects**

| Table 2: Peak voxels in respective ROIs of the BOLD contrasts (One Sample T-tests) with cluster size (k) and statistics (FWE-corrected). | | | | | | | | |
| --- | --- | --- | --- | --- | --- | --- | --- | --- |
| Contrast | Structure | Side | x | y | z | k | **T**_max_ | **P**_corr_ |
| sexual – neutral | Amygdala | L | -18 | -4 | -12 | 169 | 7.41 | <.001 |
|  |  | R | 20 | -2 | -12 | 148 | 5.09 | <.001 |
|  | OFC | L | -4 | 42 | -4 | 1160 | 9.53 | <.001 |
|  |  | R/L | 0 | 44 | -6 | 825 | 7.94 | <.001 |
|  | Insula | L | -38 | 12 | -8 | 692 | 8.89 | <.001 |
|  |  | R | 40 | 12 | -6 | 614 | 8.80 | <.001 |
| neutral – sexual | Insula | L | -36 | -18 | 18 | 78 | 8.21 | <.001 |
|  |  | R | 38 | -14 | 16 | 102 | 6.95 | <.001 |

**3.3 fMRI regression with HTC**

No whole brain or ROI results were found for the sexual – neutral contrast in association with HTC.

**4. Functional Amygdala Connectivity and HTC**

Investigating emotion neurocircuitry, functional connectivity between the amygdala and prefrontal regions is regarded as an important factor for emotion regulation (Kim *et al.*, 2011). In a model of divergent effects of progesterone and testosterone on amygdala/prefrontal connectivity, testosterone has been associated with difficulties in emotion regulation as well as with reduced amygdala/prefrontal coupling (van Wingen *et al.*, 2011) during passive emotion processing tasks. Specifically, testosterone was linked with reduced amygdala/vmPFC coupling (Stanton *et al.*, 2009) as well as with reduced amygdala/OFC coupling (van Wingen *et al.*, 2010). These findings suggest that testosterone attenuates the neural coupling between the amygdala and prefrontal areas.
We hypothesized that HTC levels are negatively linked with amygdala/OFC connectivity when viewing emotional compared to neutral pictures.

We conducted a psycho-physiological-interaction analysis (PPI; Friston *et al.*, 1997) to determine task-dependent coupling of the amygdala with the OFC. We used the left and right amygdala (threshold 50) mask from the ‘Harvard-Oxford cortical and subcortical structural atlases’ (HOC) provided by the Harvard Center for Morphometric Analysis to construct the volumes of interest (VOI). The first eigenvariate was extracted from the amygdala VOI as implemented in SPM12. The interaction term was then created by multiplying the extracted signal with the contrast of interest (negative - neutral, positive – neutral, and emotional - neutral respectively) for each subject. This interaction vector, as well as the task regressor, the extracted first eigenvariate, the task-regressors (negative, positive, sexual, neutral and rating), the movement parameters and the outlying volumes were entered into first level analyses. Functional connectivity was defined as a correlation of the psycho-physiological-interaction term in the seed region amygdala and the hemodynamic response in a ROI. Regression analyses were performed with the interaction term to investigate the association of testosterone with amygdala coupling and decoupling.

No associations of HTC and functional amygdala connectivity during emotional picture processing were found.

The lack of an association of HTC with amygdala/OFC connectivity could be explained by the passive picture perception paradigm. While connectivity effects have been found in some cases using passive viewing (Stanton et al., 2009) or emotion matching tasks (van Wingen et al., 2010), a more active explicit emotion regulation task (Banks et al., 2007; Morawetz et al., 2017) might be needed to elicit a strong enough functional connectivity effect to find associations with HTC.
